# 

**Published:** 1893-11-04

**Authors:** 


THE HOSPITAL. NOVEMBER 4TH

PRICE TWOPENCE.

No. 371, Vol. XV.—Nov. 4th, 1893.

[Registered at the General Post Office as a Newspaper for transmission in the United Kingdom and Abroad.]

WITH EXTRA NURSING SUPPLEMENT
Per Annum, 8s. 6d., post free.
Monthly Parts, 6d.; post free, 8d.
Ditto per Annum, 8s., post free.

### THE HOSPITAL

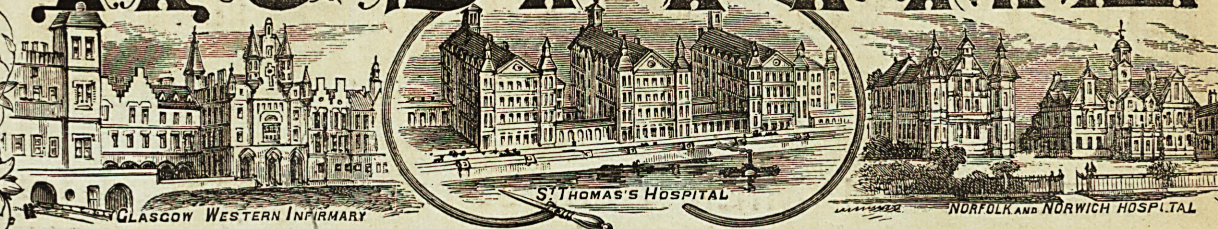

WITH EXTRA NURSING SUPPLEMENT.

No. 371. Vol. XV. SATURDAY, NOVEMBER 4TH, 1893. [TWOPENCE.

